# Supplementary material for: Biochemical and molecular characterization of the SBiP1 chaperone from Symbiodinium microadriaticum CassKB8 and light parameters that modulate its phosphorylation
Source: PLoS One. 2023 Oct 20;18(10):e0293299. doi: 10.1371/journal.pone.0293299 (PMC10588850; doi:10.1371/journal.pone.0293299)
Supplement: S1 Table — (DOCX) [file pone.0293299.s001.docx]

**S1 Table**

| Name | Sequence (5’-3’) | Length (nt) | Tm (°C) |
| --- | --- | --- | --- |
| Hsp75_50 Fw | GAG TGT TTT TGC GGC AGT CTG | 21 | 58 |
| Hsp75_500 Rv | CTC TTG TCC ACG ATC TTG TAT G | 22 | 64 |
| SmicBiP Prom F | CTC TGT GTC TTA TCC TGG CAT TCC | 24 | 64 |
| SmicBiP Prom R | CGA TCT TCT TGT CCT CTT CTT TGG | 24 | 64 |
| Hsp75STOP_BH1 Rv | CGG GAT CCC GTT ACA GCT CGT CGT GTG CC | 29 | 64 |
| DinoSL | CCG TAG CCA TTT TGG CTC AAG | 21 | 60 |
| Hsp75_Rv 50 | GGC TTC GCA GCC TGT AAT AC | 20 | 60 |
| Hsp75_F6 | CCA TCA ATC CTA CTC AGA CC | 20 | 60 |
| Hsp75_F3 | GAG GAG TTC GCG GAT GAG GAC AAG AAG G | 28 | 70 |
| Hsp75_1800 Rv | CAT GGA GTG AAT GTA GCC ATC | 21 | 60 |
