# Supplementary material for: Biochemical and molecular characterization of the SBiP1 chaperone from Symbiodinium microadriaticum CassKB8 and light parameters that modulate its phosphorylation
Source: PLoS One. 2023 Oct 20;18(10):e0293299. doi: 10.1371/journal.pone.0293299 (PMC10588850; doi:10.1371/journal.pone.0293299)
Supplement: S1 Fig — The sequence was annotated with accession number OP429595. (PDF) [file pone.0293299.s002.pdf]

S1Fig.

```
     atg tgg aaa gta gcc ttt gtg acc gtt ttt gct ctt gcg tcc ttc gcg gcc gcc aaa gaa
  1   M   W   K   V   A   F   V   T   V   F   A   L   A   S   F   A   A   A   K   E    20
     gag gac aag aag atc gat ggc cca gtg atc gga atc gac tta ggc acc acg tat tcg tgc
 21   E   D   K   K   I   D   G   P   V   I   G   I   D   L   G   T   T   Y   S   C    40
     gtg ggc atc aac aag aat gga cga gta gag att att ccg aac gat caa ggc aat cgt atc
 41   V   G   I   N   K   N   G   R   V   E   I   I   P   N   D   Q   G   N   R   I    60
     acg ccc tct tac gtg gcc ttt acc gac gac gag cgt ctg atc gga gag gcg gcg aag aat
 61   T   P   S   Y   V   A   F   T   D   D   E   R   L   I   G   E   A   A   K   N    80
     cag gcc acc atc aat cct act cag acc ctc ttc gac gtg aag cgc ctc atc ggc cgc cgc
 81   Q   A   T   I   N   P   T   Q   T   L   F   D   V   K   R   L   I   G   R   R   100
     ttt aag gac tcc acg gtg cag aag gac att aag ctc ctt cca tac aag atc gtg gac aag
101   F   K   D   S   T   V   Q   K   D   I   K   L   L   P   Y   K   I   V   D   K   120
     agc aca aag ccc atg atc gct gtg aaa gtg aag ggt gag gag aag gtc atg gct cca gaa
121   S   T   K   P   M   I   A   V   K   V   K   G   E   E   K   V   M   A   P   E   140
     gag gtg tcc tcc atg gtg ctc acc aag atg aag gag act gcc gag aac tac ttg ggc aag
141   E   V   S   S   M   V   L   T   K   M   K   E   T   A   E   N   Y   L   G   K   160
     gag gtc aag cat gcc gtc gtg acg gtg ccc gcc tat ttc aac gat gca cag cga cag tcc
161   E   V   K   H   A   V   V   T   V   P   A   Y   F   N   D   A   Q   R   Q   S   180
     acg aag gat gca ggc aca att gct ggc ctc aat gtc ctt cgt atc atc aac gag ccc acg
181   T   K   D   A   G   T   I   A   G   L   N   V   L   R   I   I   N   E   P   T   200
     gcc gca gcc atc gca tat ggt ctg gac aag aag acc gag aag aac atc ctc gtg tac gac
201   A   A   A   I   A   Y   G   L   D   K   K   T   E   K   N   I   L   V   Y   D   220
     ctt ggc ggt gga acc ttc gat gtc tcc ctc ctc acc att gac aac ggc gtc ttt gag gtg
221   L   G   G   G   T   F   D   V   S   L   L   T   I   D   N   G   V   F   E   V   240
     gtg gcc acc aac ggc gac acc cac ttg ggt gga gaa gac ttc gac cag cgt gtg atg caa
241   V   A   T   N   G   D   T   H   L   G   G   E   D   F   D   Q   R   V   M   Q   260
     cac ttc atg aag atc ttc gag aag aag cat ggc aag gac atg tcc aag gac aag cgt tcc
261   H   F   M   K   I   F   E   K   K   H   G   K   D   M   S   K   D   K   R   S   280
     atc cag aag ctg cgc agg gag gtg gag aag acc aag cga gcg ctg agc tcc act cac cag
281   I   Q   K   L   R   R   E   V   E   K   T   K   R   A   L   S   S   T   H   Q   300
     gca cgc ctg gag att gag gcc ctc tac gat ggt act gac ttc tct gag aca ctg acc cgc
301   A   R   L   E   I   E   A   L   Y   D   G   T   D   F   S   E   T   L   T   R   320
     gct cgc ttt gag gag ctg aac gcc gac ctc ttc aag aac acc ctt ggc ccc gtg aag cag
321   A   R   F   E   E   L   N   A   D   L   F   K   N   T   L   G   P   V   K   Q   340
     gtg ttg gac gac tca ggc ctg aag aag aac cag gtc gac gag atc gtg ttg gtg ggc ggc
341   V   L   D   D   S   G   L   K   K   N   Q   V   D   E   I   V   L   V   G   G   360
     tcc acc cga att ccc aag gtg cag cag ctg atc aag gac ttc ttc aat ggc aag gag ccc
361   S   T   R   I   P   K   V   Q   Q   L   I   K   D   F   F   N   G   K   E   P   380
     aac cgc ggc att aac ccc gac gag gcc gtg gcg tac ggc gct gcc gtg cag gct ggt att
381   N   R   G   I   N   P   D   E   A   V   A   Y   G   A   A   V   Q   A   G   I   400
     ctg agt ggt gaa ggt ggc cag gat ttg ctg ctt ttg gac gtg acg cca ttg acc ctg gga
401   L   S   G   E   G   G   Q   D   L   L   L   L   D   V   T   P   L   T   L   G   420
     atc gag acc gtg ggc ggt gtc atg acc aag ctg atc tcc cgc aac aca gtg atc ccg aca
421   I   E   T   V   G   G   V   M   T   K   L   I   S   R   N   T   V   I   P   T   440
     aag aag agt cag atc ttc tcc act tac cag gac aac cag cct gcg gtg aac atc cag gtc
441   K   K   S   Q   I   F   S   T   Y   Q   D   N   Q   P   A   V   N   I   Q   V   460
     ttc gag ggc gag cga ccc atg acc aag gac aac cac ctg ctg gga aag ttt gag ctg ggt
461   F   E   G   E   R   P   M   T   K   D   N   H   L   L   G   K   F   E   L   G   480
     gga atc ccc cct gct cct cgt gga cag ccc cag atc gag gtg act ttc gag atc gac tcc
481   G   I   P   P   A   P   R   G   Q   P   Q   I   E   V   T   F   E   I   D   S   500
     aac ggt atc ctg aat gtg ggc gca gag gac aag ggc acc ggc aag agc gag aag att acc
501   N   G   I   L   N   V   G   A   E   D   K   G   T   G   K   S   E   K   I   T   520
     atc aca aac gac aag ggc agg ctg acc gaa gag cag atc gag aag atg atc cgc gaa gcc
521   I   T   N   D   K   G   R   L   T   E   E   Q   I   E   K   M   I   R   E   A   540
     gag gag ttc gcg gat gag gac aag aag gtc aag gag cgc gtg gac gcc aag aat gct ttc
541   E   E   F   A   D   E   D   K   K   V   K   E   R   V   D   A   K   N   A   F   560
     gat ggc tac att cac tcc atg cga tct gct aca gag ggc tct gga gac aac aag ggc ctc
561   D   G   Y   I   H   S   M   R   S   A   T   E   G   S   G   D   N   K   G   L   580
     agt gag aag atg gac tcc gat gag aag gag aag att cta gat gcc cta aag gat ggc cag
581   S   E   K   M   D   S   D   E   K   E   K   I   L   D   A   L   K   D   G   Q   600
     tct tgg ctg gac tcc aac ccg gag gca gat gct gag gag atc aag gag aag cac aag gag
601   S   W   L   D   S   N   P   E   A   D   A   E   E   I   K   E   K   H   K   E   620
     gtc gag ggc att tgc gcg ccc atc gtc tcc aag tac tac ggc ggc ggt ggt gcc ggc ggt
621   V   E   G   I   C   A   P   I   V   S   K   Y   Y   G   G   G   G   A   G   G   640
     gct ggc ggc gca gac gac gac gag gag gag gca cac gac gag ctg taa
641   A   G   G   A   D   D   D   E   E   E   A   H   D   E   L   *                   655
```
