# Supplementary figures and images for: Biochemical and molecular characterization of the SBiP1 chaperone from Symbiodinium microadriaticum CassKB8 and light parameters that modulate its phosphorylation
Source: PLoS One. 2023 Oct 20;18(10):e0293299. doi: 10.1371/journal.pone.0293299 (PMC10588850; doi:10.1371/journal.pone.0293299)

S2Fig.

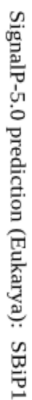

Supplement: S2 Fig — According to the software, there is a 0.998 likelihood for a cleavage site between Arg18 and Lys19 (green dashed line) for SPase I (Sec/SPI) (red continuous line). (PDF) [file pone.0293299.s003.pdf]

S4Fig.

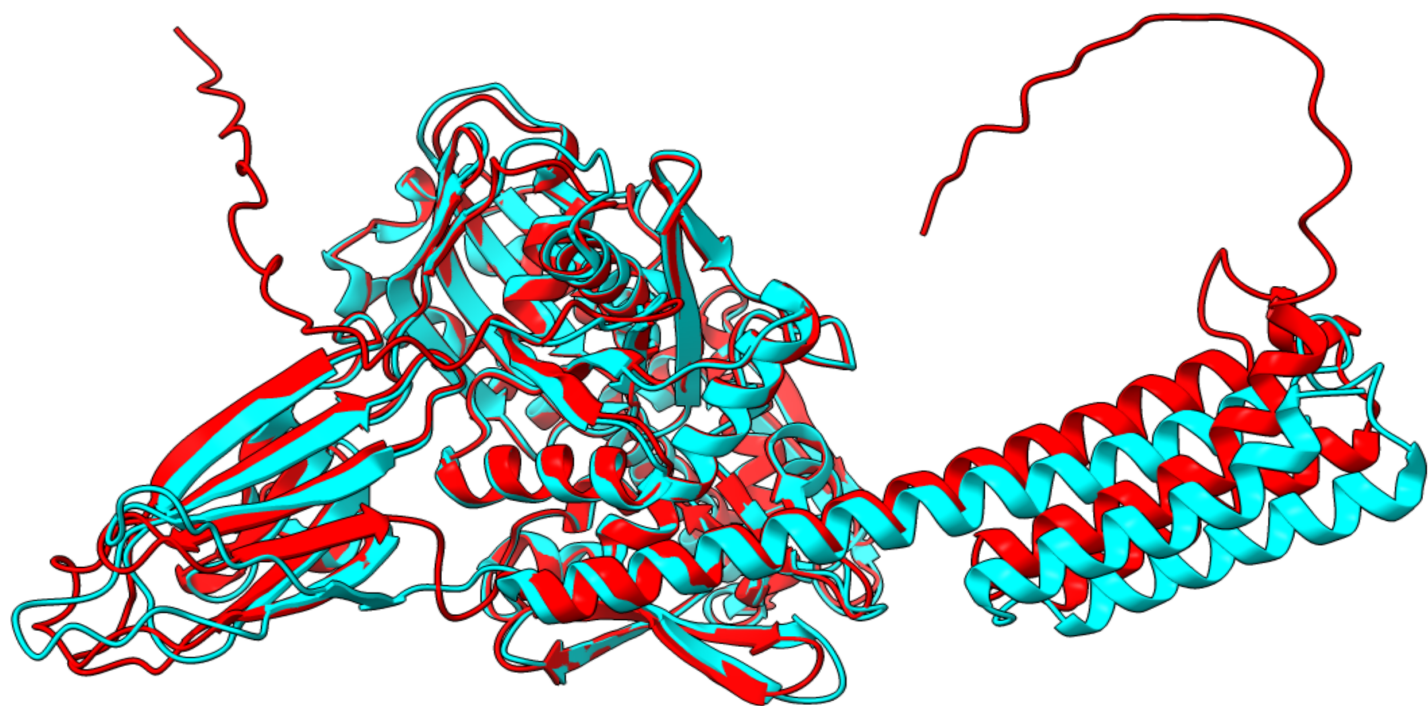

Supplement: S4 Fig — The SBiP1 3D structure obtained by AlphaFold2 (red) was superimposed on the 3D structure of H. sapiens BiP (cyan; PDB: 5e84). A close structural homology between both 3D structures is observed. (PDF) [file pone.0293299.s005.pdf]

S5Fig.

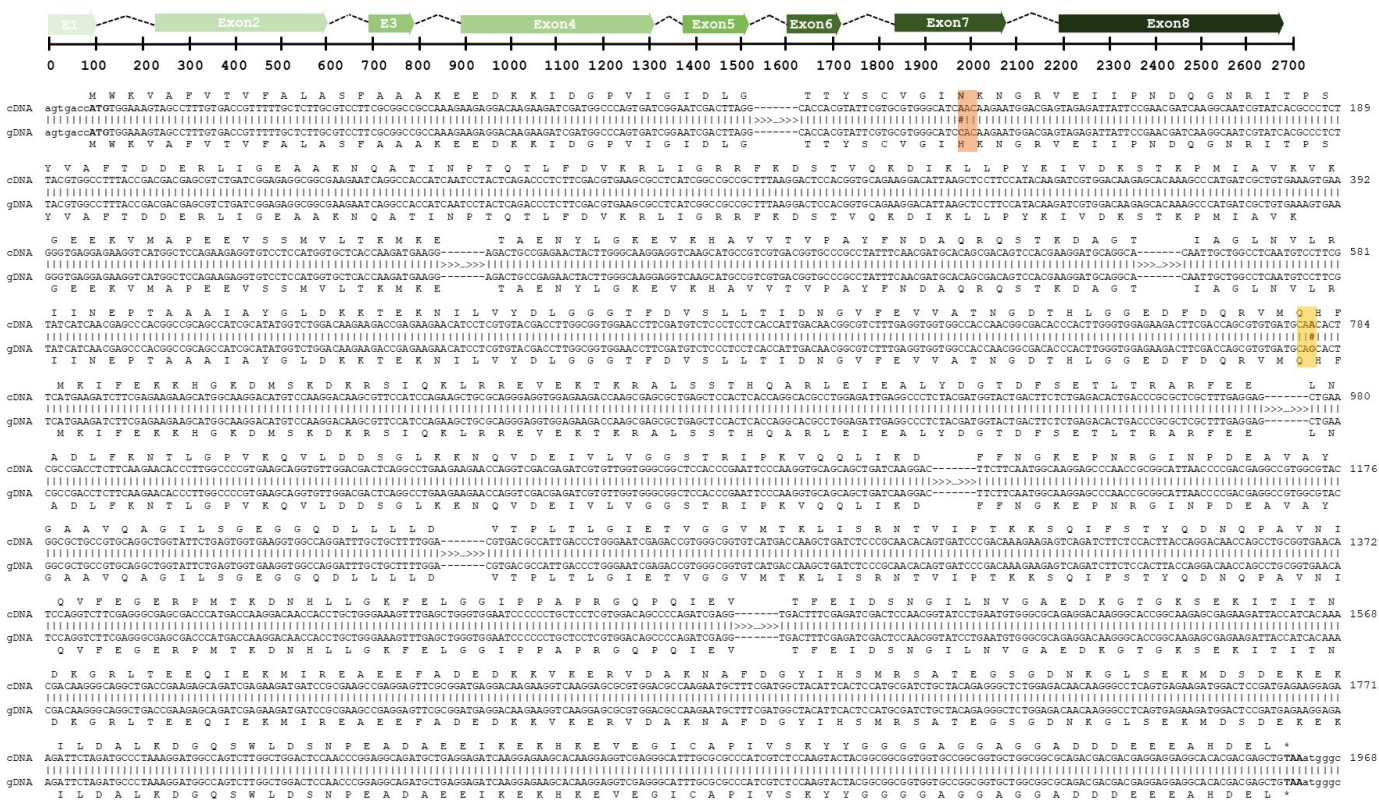

Supplement: S5 Fig — On top, schematic representation of intron/exon organization of the SBiP1 gene. On the bottom, the coding sequence of the SBiP1 gene (cDNA) compared to the reported (25) genomic sequence (gDNA). Nucleotides are numbered according to the cDNA sequence; the start and stop codons are shown in boldface, and introns are shortened within the >>>….>>> characters. The amino acid sequence is displayed in one-letter code. We found one substitution (A130C, shaded in orange) that changed the His44 for an Asn, and one synonymous substitution (A780G, Gln260Gln, shaded in yellow). (PDF) [file pone.0293299.s006.pdf]

S6Fig.

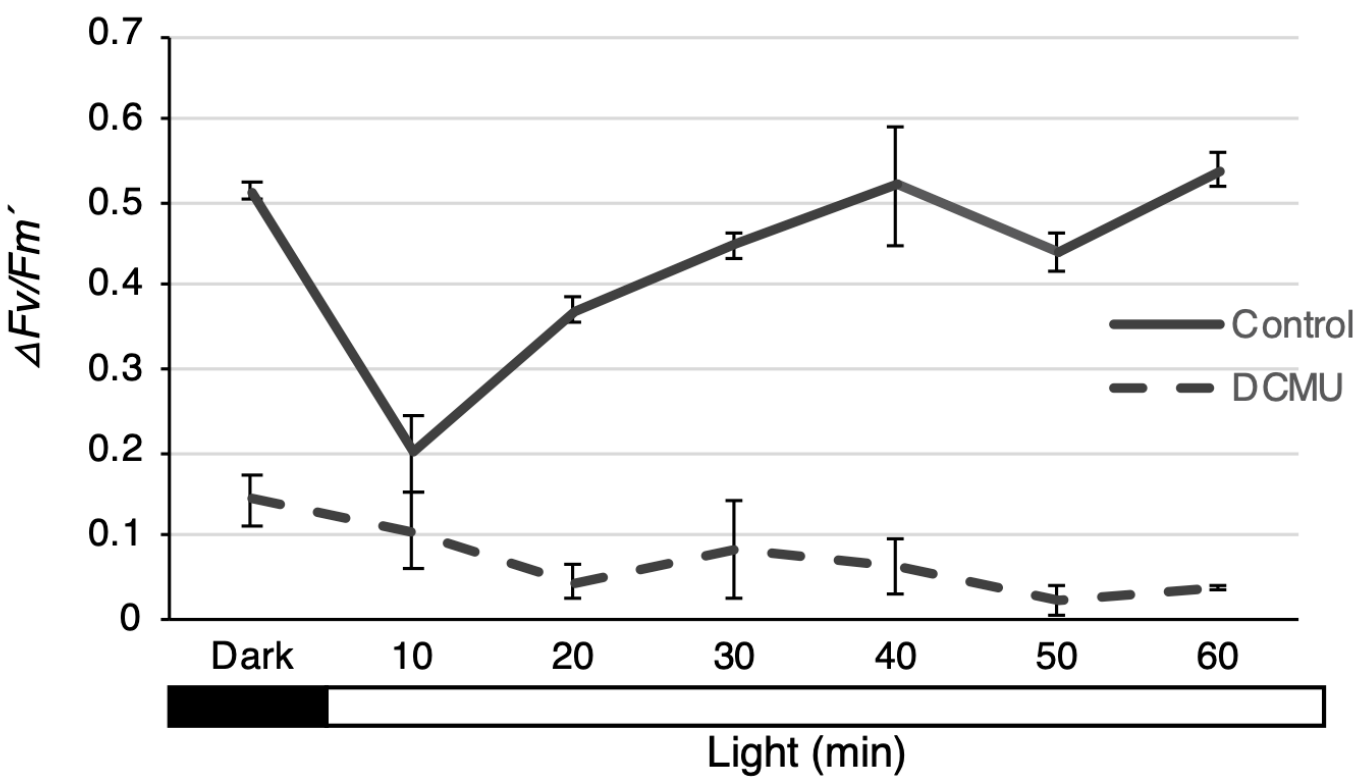

Supplement: S6 Fig — CassKB8 PSII photochemical efficiency with (dotted line) or without (continuous) DCMU from 2 h before the transition from dark to light and up to the first hour after the light onset (schematically depicted with the black/white lower bar. The cells without DCMU show the expected behavior of increase in the photochemical conversion with light whereas those with DCMU show constant low values throughout. Measurements show the average of three biological replicates. (PDF) [file pone.0293299.s007.pdf]
