# Supplementary material for: Biochemical and molecular characterization of the SBiP1 chaperone from Symbiodinium microadriaticum CassKB8 and light parameters that modulate its phosphorylation
Source: PLoS One. 2023 Oct 20;18(10):e0293299. doi: 10.1371/journal.pone.0293299 (PMC10588850; doi:10.1371/journal.pone.0293299)
Supplement: S3 Fig — All highlighted in black boxes: two predicted phosphorylation sites, a Tyr residue at position 309, and an experimentally demonstrated highly conserved Thr residue (T513); in addition, Arg residues at position 465 and 487 represent putative ADP-ribosylation sites. Positions are numbered according to the Symbiodinium microadriaticum CassKB8 SBiP1 sequence. (PDF) [file pone.0293299.s004.pdf]

S3Fig.

Organism	Y309	T513	R465 … R487
Cucumis sativus	VRVEIESLFDGTDFSEP	VKAEDKGTGKSEKIT	FEGERSLTKDCRNLGKFDLTGIPPAPRGTPQI
Camelia lanceoleosa	IRVEIESLFDGTDFSEP	VKAEDKGTGKSEKIT	FEGERSLTKDCRLLGNFDLSGIPPAPRGTPQI
Arabidopsis thaliana	VRVEIESLFDGVDLSEP	VKAEDKASGKSEKIT	FEGERSLTKDCRLLGKFDLTGVPPAPRGTPQI
Symbiodinium pilosum	ARLEIEALFDGVDFSET	VAAQDKGTGRTEKIT	HEGERAMTKDNHRLGNFDLR-VPPAPRGQPQI
Crypthecodinium cohnii	ARLEIEALFDGVDFSET	VGAEDKATGKGEKIT	FEGERPMTKDNHLLGKFELGGIPPAPRGQPQI
Oryza sativa	VRVEIESLFDGTDFSEP	VKAEDKGTGKSEKIT	FEGERSMTKDCRLLGKFDLSGIPAAPRGTPQI
Solanum tuberosum	VRVEIESLFDGVDFSEP	VKAEDKASGKSEKIT	FEGERSMVKDCRLLGKFDLTGIPPAPRGTPQI
Zea mays	VRVEIESLFDGTDFSEP	VKAEDKGTGKSEKIT	FEGERSMTKDCRLLGKFDLNGIPSAPRGTPQI
Triticum aestivum	VRVEIESLFDGTDFSEP	VKAEDKGTGKSEKIT	FEGERSMTKDCRLLGKFDLSGIPPAPRGTPQI
Plasmodium falciparum	ATIEVDSLFDGIDYNVN	VTALDKGTGKQNQIT	YEGERAMTKDNNLLGKFQLEGIPPAPRSVPQI
Trypanosoma cruzi	ARVEVDSLTEGFDFSEK	VSAVDKSSGKKEEIT	YEGERQMTKDNRLLGKFELSGIPPAPRGVPQI
Danio rerio	ASIEIDSLYEGIDFYTS	VSAADKSTGKQNRIT	FEGERAMTKDNNLLGKFELTGIPPAPRGVPQI
Chlamydomonas reinhardtii	VRVEIEALYEGIDLSEP	VAAEDKGTGKKEKIT	YEGERAMTKDNHKLGQFDLNGIPPAPRGTPQI
Symbiodinium microadriaticum CassKB8	ARLEIEALYDGTDFSET	VGAEDKGTGKSEKIT	FEGERPMTKDNHLLGKFELGGIPPAPRGQPQI
