# Supplementary material for: Biochemical and molecular characterization of the SBiP1 chaperone from Symbiodinium microadriaticum CassKB8 and light parameters that modulate its phosphorylation
Source: PLoS One. 2023 Oct 20;18(10):e0293299. doi: 10.1371/journal.pone.0293299 (PMC10588850; doi:10.1371/journal.pone.0293299)
Supplement: Attachment — Submitted filename: Response Lett.pdf [file pone.0293299.s008.pdf]

UNIVERSIDAD NACIONAL AUTÓNOMA DE MÉXICO

INSTITUTO DE CIENCIAS DEL MAR Y LIMNOLOGÍA
UNIDAD ACADÉMICA DE SISTEMAS ARRECIFALES

DIRECCIÓN
PROLONG. AV. NIÑOS HÉROES S/N
77580 PUERTO MORELOS, QUINTANA ROO, MÉXICO

APARTADO POSTAL 13
77500 CANCÚN, Q. ROO; MÉXICO

October 4, 2023

Dear Reviewers:

Thank you very much for your careful review and incisive comments which will definitely and substantially improve our work entitled “**Biochemical and molecular characterization of the SBiP1 chaperone from *Symbiodinium microadriaticum* CassKB8 and light parameters that modulate its phosphorylation**” by Raúl Eduardo Castillo-Medina, Tania Islas-Flores, Estefanía Morales-Ruiz and Marco A. Villanueva.

We here provide the point-by-point response to your queries as follows:

Reviewer No. 1

1. The first section in Results: the reason for the sequence correction should be explained before reporting the correct ORF.

Response: We have modified the text to mention the reason for sequence correction before reporting the correct ORF (lines 272-277).

2. Line 267: The result of SignalP signal peptide prediction should be given in detail (the resulting figure of SignalP, the values of possibility, or other predicted parameters).

Response: A supplementary figure S2 with the results has been added and a brief description mentioned in the methods (lines 169-171 and 279).

3. Line 138: Describe the sequence analyses in a new section.

Response: The sequence analyses have been separated and are described in a new section (line 134 and 164).

4. Line 293: a useless parenthesis.

Response: The parenthesis has been deleted (line 305).

5. Line 469-471: Asn is not a basic amino acid. There is no evidence to support the sentence, and it should be removed.

Response: The line has been deleted (line 482).

Reviewer No. 2

Query: Line 29-30: This sentence confused me. Please rephrase it.

Response: It has been rewritten for clarity (lines 24-25).

Query: Line 75-77: 869 sequences protein kinases sequences are described in González-Pech et al (2017) [11] only while Supplementary Table 12 in Liu et al (2018) [12] gives a more comprehensive representation of these domains in Symbiodiniaceae. It might be helpful to express the abundance of kinase domains as a range or percentage within Symbiodiniaceae. With more Symbiodiniaceae genomes, this sentence provides larger context.

Response: The kinase domains have been expressed as a range and “sequences” has been changed to “families” as correctly reported (lines 71-72).

Query: Line 100-102: It might be helpful to readers if this sentence can be rephrased to clarify that dephosphorylation of BiP correlates with chaperone activation (Díaz-Troya S et al [15]) or authors can justify why they termed it as "presumptive".

Response: The term ”presumptive” has been deleted, the words “implying its activation” was added and the corresponding references (Díaz-Troya et al. 2011 [15] and Nitika et al. 2020 [16] were already provided in the sentence (lines 97-98).

Query: Line 167-168: Please describe briefly how the substrate binding domain and the ADP-ribosylation sites were determined.

Response: A brief description was included (lines 173-174 and 176-177).

Query: Line 213-214: How was the light intensity controlled? Was it in automated option in an incubator?

Response: A sentence with this information has been added in the corresponding Methods section (lines 226-228).

Query: Line 278-279: Please show the conservation plot of the phosphorylation residues as a supplementary figure; it can be confirmed for Thr 513 as shown in figure 5 in [15] but there is no plot for Tyr 309. The same applies for Arg 465 and 487. A simple alignment should be enough.

Response: A supplementary figure showing the alignment and a slight change in the text was made (line 290; S3 Fig. mentioned in lines 291 and 294).

Query: Line 291-293: In Fig S2 both structures are difficult to differentiate due to the color. Can the authors show one of the structures in a different color to ease visualisation.

Response: A figure with contrasting colors now S4 Fig. has replaced the former S2 (mentioned in line 304).

Query: Line 352-265: It is difficult to confirm or ascertain what authors are claiming in terms of dephosphorylation level from the blot. I urge the authors to add a statement to tone down this claim. I am glad that Line 356-369 does support this claim statistically.

Response: The statement has been toned down to say "and was still observed at apparently higher levels after 30 min of light (line 368).

Query: Line 469: Typo "synonimous"

Response: The sentence was deleted at the request of Reviewer 1 (line 482) and the same typo was corrected from line 479.

Query: Line 522: Typo "dephsophorylation"

Response: Typo was corrected to "dephosphorylation" (line 533).

Query: Line 587-589: I would encourage the authors to give a broader perspective to their discussion especially to adaptation to Symbiodiniaceae in different symbiotic relationship. In this case the strain was obtained from jellyfish. It should be possible to look at Symbiodiniaceae RNAseq data publicly available to support these observations. There is a brief mention to shall water Symbiodiniaceae (Line 544-545). The role of such post-translational events in key proteins must have been an evolutionary advantage for symbiosis especially with corals.

Response: In this regard, we chose not to enter into too much speculation on the subject mentioned by the reviewer since the experiments from our work were carried out in cultured Symbiodiniaceae. Instead, we emphasized this fact and suggested that further experimentation is required on endosymbionts from diverse holobiont systems including *C. xamachana* to be able to understand evolutionary adaptations. (lines 566-572).

Additional: We corrected "RNA" instead of "DNA" from line 135, added the methodology for genomic PCR extraction which was missing (lines 144-151), and corrected typo "is" for "are" on line 305.

We hope you will now find the work suitable for publication.

Dr. Marco A. Villanueva, on behalf of all authors.

TELS. PTO. MORELOS (998) 871-0219, 871-0009, 871.0367, 871.0584. MÉXICO (55) 562-26546, 562-226549
